# Minimising prescribing errors in the ICU

**DOI:** 10.1186/cc13191

**Published:** 2014-03-17

**Authors:** DJ Melia, S Saha

**Affiliations:** 1 Queen's Hospital, Romford, UK

## Introduction

We aimed to audit the prescribing practice on a busy 14-bedd general ICU, and develop standardised practices and tools to improve safety. Prescribing errors occur as commonly as in 10% of UK hospital admissions, costing 8.5 extra bed days per admission, and costing the National Health Service an estimated £1 billion per annum [1]. The majority of these mistakes are avoidable [2].

## Methods

We audited the daily infusion charts of all patients in three separate spot checks, over 1 week. We assessed all aspects of prescriptions that make them legal and valid, in accordance with national guidance [3]. New procedures were introduced, which included a standardised prescription sticker, with common, preprinted, infusion prescriptions on (noradrenaline, propofol, and so forth), and education on using the new prescription stickers. A month later the audit process was repeated.

## Results

**Figure 1:**
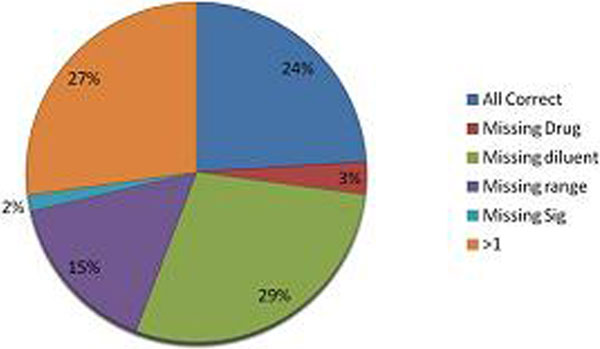
**Accuracy of prescriptions before intervention**.

**Figure 2:**
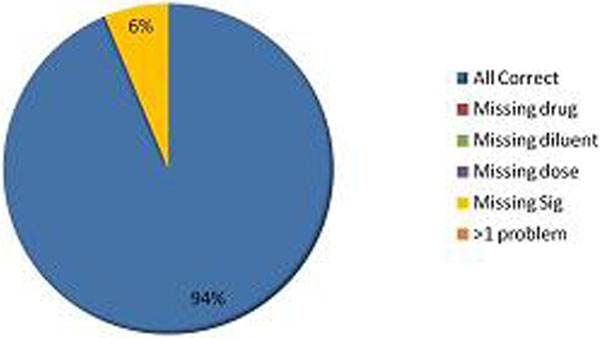
**Accuracy of prescriptions after intervention**.

We assessed 129 prescriptions in the first round, and 111 after intervention, demonstrating a 70% improvement in safe prescribing. Only 24% of prescriptions initially fulfilled best practice criteria, improving to 94% afterwards. We also reduced the number of infusions running without prescription, 7 (6%) versus 24 (19%). See Figures 1 and 2.

## Conclusion

Our audit supports the need for standardised prescribing practices within critical care, especially when dealing with potentially harmful vasoactive/sedative drugs. With a small, cost-effective intervention (£20 for 6,200 stickers), we improved prescribing accuracy, and thus patient safety in intensive care.
